# Transcriptomic Profiling Identifies Hub Transcription Factors Associated with the Protective Effects of Licochalcone A Against HFD-Induced MASLD

**DOI:** 10.3390/nu18213564

**Published:** 2026-10-28

**Authors:** Shanshan Zhao, Bohan Lei, Rongxia Li, Shuai Wang

**Affiliations:** 1 School of Medical Technology and Engineering, Henan University of Science and Technology, 263 Kaiyuan Ave, Luoyang 471023, China; 2 College of Animal Science and Technology, Henan University of Science and Technology, 263 Kaiyuan Ave, Luoyang 471023, China

**Keywords:** metabolic dysfunction-associated steatotic liver disease (MASLD), nonalcoholic fatty liver disease (NAFLD), Licochalcone A (LA), transcription factors, network analysis

## Abstract

Background/Objectives: Metabolic dysfunction-associated steatotic liver disease (MASLD, formerly NAFLD) lacks approved specific pharmacological treatments and exhibits complex pathogenesis. Licochalcone A (LA), a flavonoid extracted from *Glycyrrhiza glabra*, possesses anti-inflammatory and lipid-modulating activities, yet its protective effects and transcriptional regulatory mechanisms against MASLD remain poorly defined. Methods: We established an HFD-induced MASLD mouse model to assess the in-vivo protective effect of LA. Hepatic RNA-sequencing was performed to profile transcription factor (TF) expression and identify differentially expressed transcription factors (DE-TFs). Functional enrichment and protein–protein interaction (PPI) network analysis were conducted to characterize DE-TFs and screen hub TFs. RT-qPCR was used to validate the expression patterns of identified hub TFs. Results: LA administration attenuated hepatic lipid deposition in HFD-fed MASLD mice. In total, 149 DE-TFs were identified, including 95 DE-TFs from the comparison of HFD versus control mice and 122 DE-TFs from LA-treated mice versus HFD mice, with 68 DE-TFs shared between the two comparisons. Functional enrichment indicated that these DE-TFs were associated with RNA polymerase II-dependent transcription, nuclear components, DNA binding, signal transduction, and metabolic-disease-related pathways. Nine hub TFs (*Myc*, *Fos*, *Runx1*, *Mef2c*, *Spi1*, *Sox9*, *E2f1*, *Cebpa*, and *Pparg*) were screened via PPI analysis. Seven hub TFs were up-regulated, while *Cebpa* and *Pparg* were down-regulated in MASLD mice; LA intervention partly reversed this dysregulated expression profile, as further verified by RT-qPCR. Conclusions: LA mitigates the development of HFD-provoked MASLD-associated hepatic lesions and is associated with partially restored transcript levels of aberrantly expressed hub TFs in mice. This work provides transcriptome-level resources for understanding LA-mediated hepatoprotection against HFD-induced MASLD development and highlights candidate TF targets relevant to MASLD research.

## 1. Introduction

Metabolic dysfunction-associated steatotic liver disease (MASLD), formerly known as nonalcoholic fatty liver disease (NAFLD), has been recognized as the leading cause of global chronic liver disease, with a prevalence of 24–36% worldwide [1]. This chronic liver disease is predominantly characterized by metabolic dysfunction and systemic insulin resistance, including hyperglycemia, hypertension, obesity, and dyslipidemia, whose progressive course elevates the risk of liver fibrosis, cirrhosis, liver failure, and even hepatocellular carcinoma [2]. Given the complex pathophysiology of MASLD, which conforms to the multiple-hit hypothesis, there are currently no approved specific pharmacological interventions for MASLD [3,4]. Exploring bioactive natural agents that can ameliorate MASLD-related metabolic disturbances has therefore become an important focus for nutrition-related research.

Traditional Chinese medicine has unique advantages in multi-target, multi-pathway and multi-level intervention as well as holistic regulation, which makes it a promising source for identifying bioactive compounds for complex metabolic disorders with incompletely clarified pathogenesis. Licochalcone A (LA), a flavonoid compound derived from the traditional Chinese medicinal herb *Glycyrrhiza glabra*, has been reported to suppress inflammatory responses, regulate lipid metabolism and enhance liver function [5,6,7,8]. The α,β-unsaturated ketone conjugated structure and unique isopentenyl side chain of LA constitute the chemical basis for its pharmacological activities [9]. Compared with other licorice-derived flavonoids such as isoliquiritigenin, the isopentenyl modification endows LA with stronger membrane affinity and biological activity. Studies have demonstrated that LA exerts its effects by regulating PI3K, MEK1, and B-Raf [10]. Nevertheless, its direct molecular targets, mode of action, and distinct transcriptional-regulatory features differentiating it from related flavonoids remain poorly defined in in-vivo MASLD models.

Previous studies have demonstrated that transcription factor (TF) regulation plays a crucial role in hepatic lipogenesis, e.g., USF, SREBPs and ChREBP of the bHLH-LZ family and LXR belonging to the NHR superfamily [11]. FOXO can sustain metabolic and cellular homeostasis in the liver [12]. Nevertheless, the hepatoprotective effects of LA against MASLD, as well as the TFs involved in LA-mediated amelioration of MASLD-associated hepatic alterations, remain to be elucidated.

Whole-transcriptome profiling identifies abundant downstream genes but not upstream master transcriptional regulators. TFs serve as high-order regulatory nodes, where modest expression shifts remodel lipogenic, inflammatory and liver injury gene networks. We therefore prioritized TFs over the full transcriptome to uncover key regulators of MASLD and LA hepatoprotection. Prior LA/MASLD work only reported phenotypes and limited downstream genes, without systematic profiling of LA-modulated TF networks. Our working hypothesis: LA mitigates HFD-induced MASLD by restoring dysregulated core hepatic hub TFs.

In this study, we evaluated the protective effects of LA against the development of high-fat diet (HFD)-induced MASLD in mice. Using this mouse model, RNA-sequencing (RNA-seq) was performed to systematically investigate the alterations in hepatic TFs during MASLD progression and screen for differentially expressed TFs (DE-TFs) associated with the ameliorative effect of LA on HFD-provoked hepatic disturbances. These DE-TFs were further subjected to functional enrichment analysis to elucidate their biological roles. In addition, protein–protein interaction (PPI) network analysis was used to screen hub TFs whose transcript expression correlates with LA-dependent hepatoprotection.

## 2. Materials and Methods

### 2.1. Animal Treatments and Sample Collection

Four-week-old specific-pathogen-free (SPF) C57BL/6 male mice (Zhengzhou Jiacheng Biotechnology Co., Ltd., Zhengzhou, Henan, China), weighing 18–22 g, were bred in an SPF environment, with a humidity at 40–60%, temperature at 20 ± 2 °C, and an alternating light cycle for 12 h. After the adaptive feeding for 1 week, the experimental mice were randomly assigned to three groups (*n* = 6): control group, HFD group, and HFD-LA group. After acclimation, animals were grouped by random number table. Researchers for testing and data analysis were blinded to group allocation. Animals were excluded if they died before the endpoint or had sampling failure or contaminated samples.

LA (purity > 96%, S29891) was purchased from Shanghai yuanye Bio-Technology Co., Ltd. (Shanghai, China). Mice in the control group, HFD group, and HFD-LA group were respectively fed a normal chow diet, HFD, and HFD supplemented with LA (Prepared by Anlimao Biotechnology Co., Ltd., Hangzhou, China) at a formulated target dose of 15 mg/kg per day [8]. This value represents the designed dose of LA added to the diet rather than the measured actual LA intake of the mice. LA intervention was initiated simultaneously with HFD feeding. After 17 weeks, the mice were euthanized by intraperitoneal injection of pentobarbital sodium. Liver tissues were harvested from six mice per group and allocated into two subgroups. Subgroup A (*n* = 3 mice per subgroup) was used for pathological and biochemical analyses, including hepatic TG measurement, hematoxylin and eosin staining (HE) and Oil Red O staining, and RNA isolation for RT-qPCR. The other subgroup (*n* = 3 mice per subgroup) was dedicated to RNA-seq transcriptome profiling. For subgroup A, tissue aliquots for pathological sectioning were fixed in 4% paraformaldehyde. Hepatic TG concentrations were determined using commercial assay kits (Solarbio) in accordance with the manufacturer’s instructions. The remaining liver tissues were rapidly stored at −80 °C for RNA extraction.

### 2.2. Hematoxylin and Eosin Staining (HE) and Oil Red O Staining

For histological analysis, liver tissues were fixed in 4% paraformaldehyde, dehydrated with graded ethanol, cleared in xylene, and embedded in paraffin. The tissues were cut into 5 μm sections and stained with HE to observe hepatic histopathological changes. After staining, the sections were dehydrated, cleared, and mounted for microscopic examination.

Oil Red O staining was used to evaluate lipid accumulation in liver tissues. Liver tissues were harvested from identical anatomical sites, fixed in 4% paraformaldehyde, embedded in OCT compound, and cut into 8 μm frozen sections. Sections were equilibrated in 60% isopropanol at room temperature. Staining was performed with an Oil Red O Staining Kit (Solarbio, Beijing, China) following the manufacturer’s protocol. Stained slices were observed under an optical microscope. Representative images were acquired, and the lipid droplet area was quantified by ImageJ (1.46r). Histological scoring was performed in a blinded manner.

### 2.3. RNA Isolation, Library Construction and RNA-Seq

Total RNA was isolated from the liver tissues using the TRIzol reagent (Invitrogen, Carlsbad, CA, USA) according to the manufacturer’s protocol. RNA samples with RNA integrity number (RIN) ≥ 7 and 28S/18S ≥ 0.7 were further analyzed using a NanoDrop 2000 spectrophotometer (Thermo Scientific, Wilmington, DE, USA) and an Agilent 2100 Bioanalyzer (Agilent Technologies, Santa Clara, CA, USA). Subsequently, ribosomal RNA was depleted and the RNA was fragmented. Double-stranded cDNA was synthesized using the fragmented RNA as a template with hexamer random primers. The cDNA library was then constructed through purification, end repair, A-tailing, adaptor ligation, and PCR amplification. The constructed library was determined using the Agilent 2100 Bioanalyzer and then sequenced on an Illumina Novaseq 6000 platform (Illumina, San Diego, CA, USA). Library construction and RNA sequencing were conducted at OE Biotech Co., Ltd. (Shanghai, China).

### 2.4. Genomic Alignment, Quantification and Differentially Expressed Gene (DEG) Analysis

Raw sequencing reads were quality-trimmed and adaptors were removed using Trimmomatic (v0.36). Fastp (v0.23.2) was used to assess the quality of raw and trimmed reads. Clean reads were mapped to the mouse reference genome assembly GRCm39 (GCF_000001635.27) using HISAT2 (v2.2.1.0). Transcript reconstruction was performed using StringTie2 (v1.3.3b) based on uniquely mapped reads. Gene-level raw read counts were quantified by HTSeq-count (v0.9.1). Notably, the raw count matrix rather than fragments per kilobase of transcript per million fragments mapped (FPKM) values was used as input for differential expression analysis; FPKM was only calculated for descriptive visualization of gene expression levels. Differential expression analysis was performed using the DESeq2 package (v1.18.0) based on negative-binomial generalized linear models. DESeq2 performs normalization of raw read counts using the median-of-ratios method. The Benjamini–Hochberg method was adopted for multiple-testing correction to compute adjusted *q*-values. Genes satisfying the criteria of |log_2_(Fold Change)| ≥ 1 and adjusted *q*-value < 0.05 were regarded as DEGs. Detailed software parameters and reference genome information are summarized in Supplementary Table S1.

### 2.5. Identification and Family Classification of TFs and Target Gene Extraction

TF annotations and family information for *Mus musculus* were obtained from AnimalTFDB v4.0 (https://guolab.wchscu.cn/AnimalTFDB4//#/ (accessed on 1 February 2026)). NCBI gene IDs of hepatic genes detected by RNA-seq were mapped to mouse TF lists in AnimalTFDB 4.0. Transcription factors were identified and classified into families based on the database’s DNA-binding domain criteria. DE-TFs were filtered from DEGs using thresholds |log_2_FC| ≥ 1 and adjusted *q*-value < 0.05. We compared TF proportions in all detected genes and in up/downregulated DEGs to identify TF families with shifted distributions. Predicted mouse TF-target pairs were downloaded from GTRD (https://gtrd.biouml.org/ (accessed on 5 February 2026)). Targets of DE-TFs were extracted, and all pairs were kept without confidence filtering. The number of predicted target genes per TF family was summarized. These targets represent computational predictions from public databases.

### 2.6. Functional Enrichment of DE-TFs

Gene Ontology (GO, http://www.geneontology.org/ (accessed on 1 April 2026)) enrichment and Kyoto Encyclopedia of Genes and Genomes (KEGG, https://www.kegg.jp/kegg/ (accessed on 1 April 2026)) pathway enrichment analyses of DE-TFs were performed using the OECloud tools at https://cloud.oebiotech.com/task/ (accessed on 1 April 2026). All graphics were generated using R (https://www.r-project.org/ (accessed on 1 April 2026)) on the OECloud platform (https://cloud.oebiotech.com/task/ (accessed on 1 April 2026)). A *q*-value < 0.05 was defined as the threshold for significant enrichment.

### 2.7. Protein–Protein Interaction (PPI) Network Construction and Hub TF Identification

All DE-TFs were loaded into the STRING online software (https://cn.string-db.org/ (accessed on 1 April 2026), v12.5). Active interaction sources included textmining, experiments, databases, co-expression, neighborhood, gene fusion, and co-occurrence. The minimum required interaction score was set to 0.7 (high confidence). Disconnected nodes in the network were then hidden. The resulting PPI network was imported into Cytoscape software (v3.6.1). Node degree was calculated as the topological parameter using the CytoHubba plugin. The top nine DE-TFs ranked by node degree were defined as hub TFs.

### 2.8. Reverse Transcriptase Quantitative Polymerase Chain Reaction (RT-qPCR) Analysis

Total RNA was extracted from collected liver tissues with a TRIzol reagent (Solarbio, Beijing, China). Complementary DNA (cDNA) was subsequently synthesized via the PerfectStart^®^ Uni RT&qPCR Kit (TransGen, Beijing, China) following manufacturer-provided protocols. RT-qPCR was carried out by using 2 × Universal SYBR Green Fast RT-qPCR Mix (ABclonal, Wuhan, China), with the expression level of *glyceraldehyde-3-phosphate dehydrogenase* (*Gapdh*) as an internal reference gene. The 2^−ΔΔCt^ method was used to calculate the relative expression of each gene in three biological replicates. The primers designed in the study are shown in Supplementary Table S2.

### 2.9. Statistical Analysis

Statistical analyses were performed using GraphPad Prism 8.0.1 (GraphPad Software Inc., San Diego, CA, USA). Ordinary one-way ANOVA and Tukey’s multiple comparisons test were performed. Data are presented as mean ± standard deviation (SD). Error bars denote SD. Differences with a Tukey-adjusted *p* value < 0.05 were regarded as statistically significant.

## 3. Results

### 3.1. Alleviation of Hepatic Lipid Accumulation by LA in MASLD Mice

**Figure 1:**
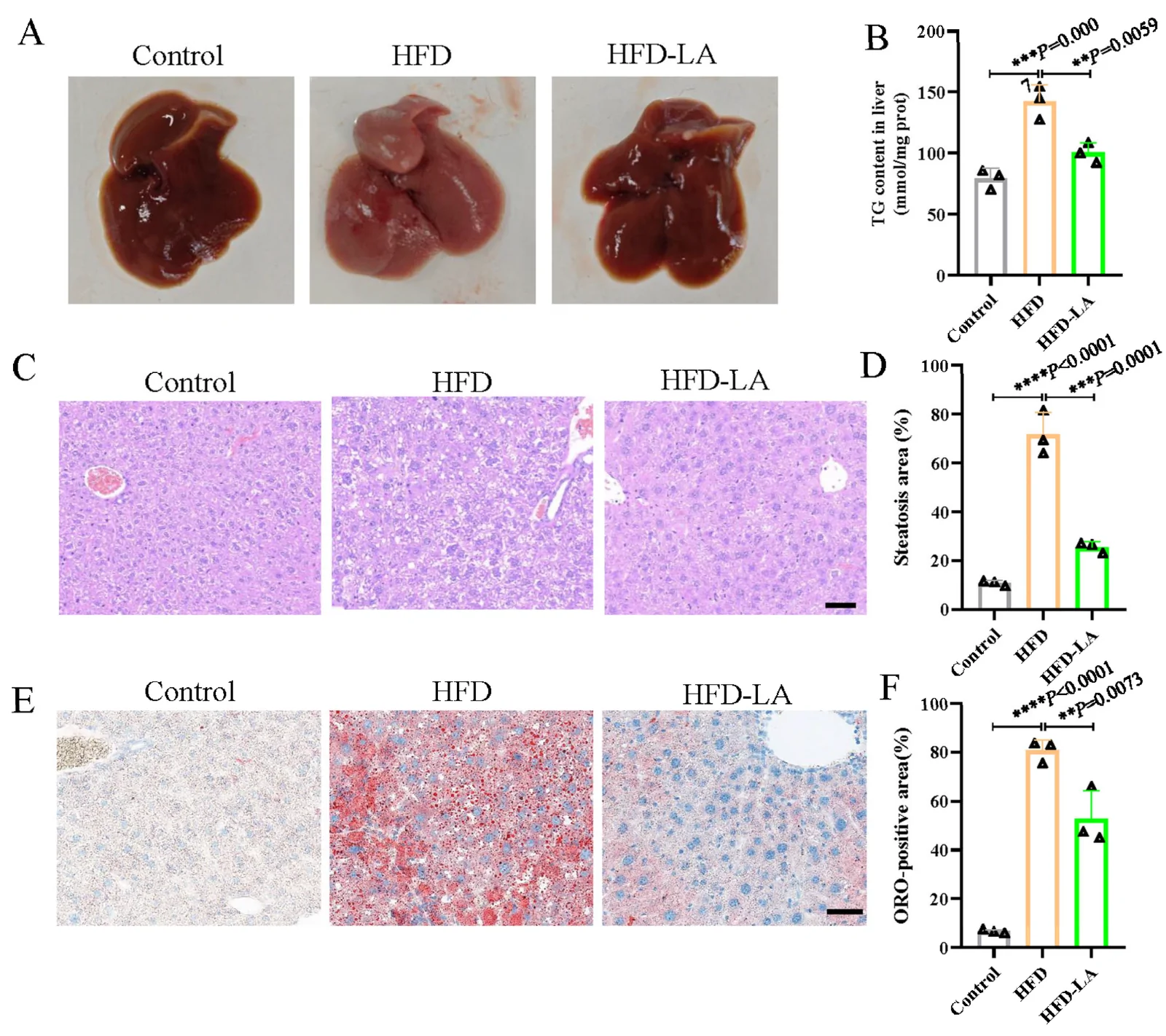
Alleviation of HFD-induced MASLD in mice by LA. (**A**) Gross morphological characteristics of the liver. (**B**) Hepatic TG content in mouse liver tissues (*n* = 3 biological replicates). (**C**,**D**) Hematoxylin and eosin staining of liver tissues. Scale bar = 50 μm. *n* = 3 biological replicates. (**E**,**F**) Oil Red O staining of liver tissues. Scale bar = 50 μm. *n* = 3 biological replicates. ** *p* < 0.01, *** *p* < 0.001, **** *p* < 0.0001.

To evaluate the hepatoprotective effects of LA against the development of HFD-induced MASLD, mice were subjected to 17-week HFD feeding with concurrent LA administration. Compared with the control group, HFD-fed mice developed enlarged, soft, pale and greasy livers, accompanied by elevated hepatic TG content; LA intervention markedly inhibited this HFD-triggered hepatic TG accumulation (Figure 1A,B). HE staining revealed macrovesicular and microvesicular steatosis in the cytoplasm of hepatocytes (Figure 1C,D). Oil-red-O staining revealed massive lipid droplet accumulation in hepatocytes, and LA intervention markedly alleviated hepatic lipid droplet deposition in mice with HFD-induced MASLD (Figure 1E,F). These results indicate that LA can partially rescue HFD-induced damage to hepatic morphological architecture and mitigate hepatic lipid accumulation in HFD-fed MASLD mice.

### 3.2. Overview of Transcriptome

A total of 214.68 M clean data was obtained from RNA samples of nine mouse livers, with a valid data ratio between 96.29% and 98.1%. The Q30 was ≥97.58%, and the GC content varied between 47.86% and 49.37%. The mapping rate of the reads aligned to the mouse genome ranged from 99.08% to 99.59% (Table 1). The FPKM expression levels in each sample are shown in Supplementary Figure S1A. The number of genes located in the FPKM ranges of 0–0.25, 1–10, and ≥10 was relatively high, whereas that in the ranges of 0.25–0.5 and 0.5–1 was relatively low. Correlation analysis among samples revealed favorable biological reproducibility within each group. The average correlation coefficients of the HFD group versus control group (HFD_VS_Control) and LA-treated group versus HFD group (HFD-LA_VS_HFD) were 0.924 and 0.934, respectively. The correlation coefficient between the control group and the HFD-LA group was considerably higher, at 0.971 (Supplementary Figure S1B). This transcriptome-level observation indicates higher global similarity in gene expression profiles between the LA-treated and control groups, yet it cannot independently demonstrate full transcriptional recovery.

### 3.3. Identification and Classification of DE-TFs

**Figure 2:**
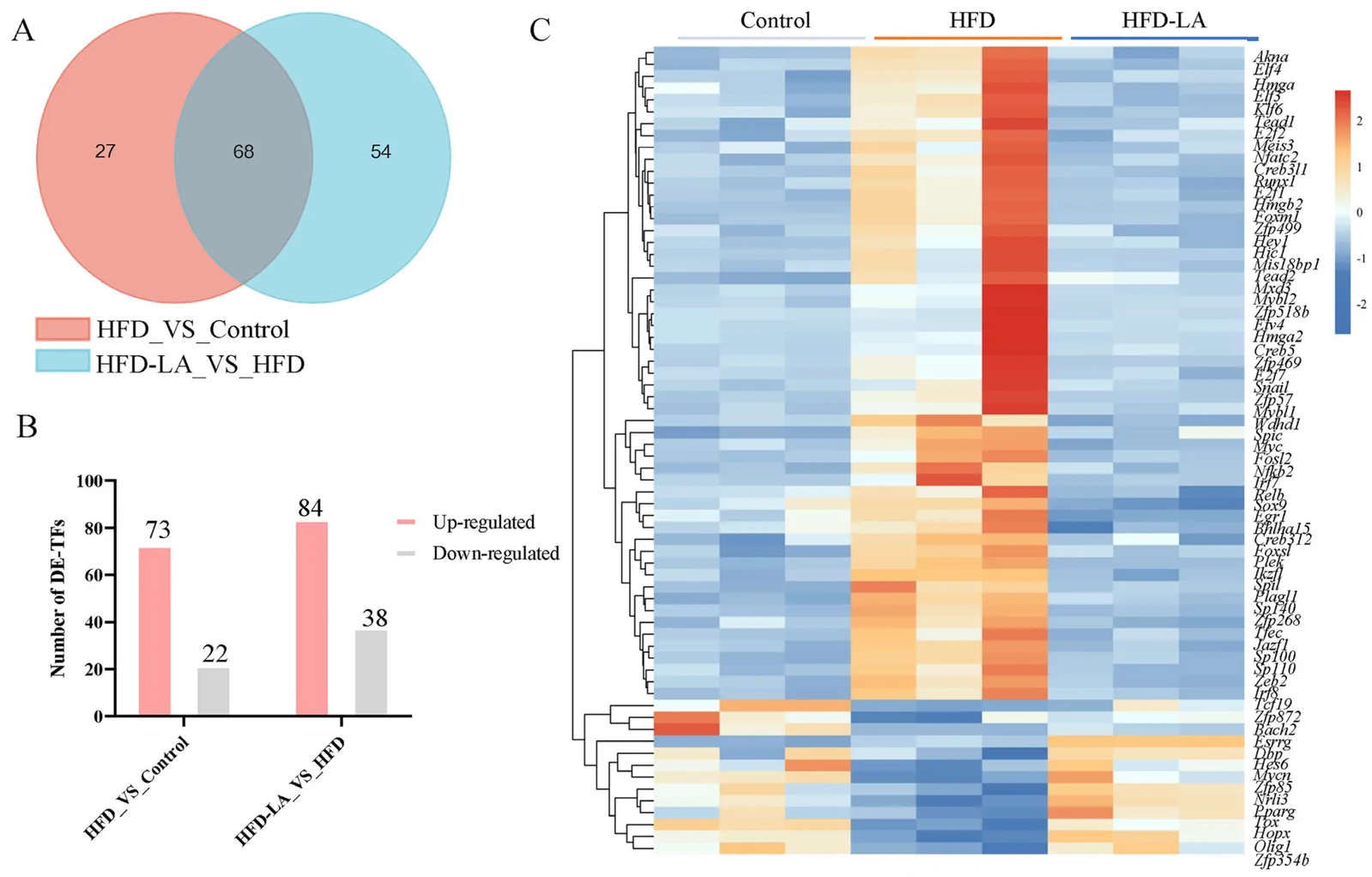
Identification and hierarchical clustering heatmaps of DE-TFs in mouse liver tissues. (**A**) Identification of unique and shared DE-TFs. (**B**) Number of DE-TFs. (**C**) Hierarchical clustering heatmap of shared DE-TFs between HFD_VS_Control and HFD-LA_VS_HFD.

As shown in Figure 2A,B, a total of 149 DE-TFs were identified, among which 95 (73 up-regulated and 22 down-regulated) and 122 (38 up-regulated and 84 down-regulated) DE-TFs were respectively detected in HFD_VS_Control and HFD-LA_VS_HFD, with 68 DE-TFs shared between the two comparisons. Furthermore, cluster analysis revealed the clear separation of TFs in each comparison. Notably, among the 68 shared TFs, 67 showed opposite expression directions upon HFD-LA treatment relative to the HFD-induced changes, while one TF (Dbp) was further upregulated (Figure 2C, Supplementary Table S3 and Supplementary Figure S2).

**Figure 3:**
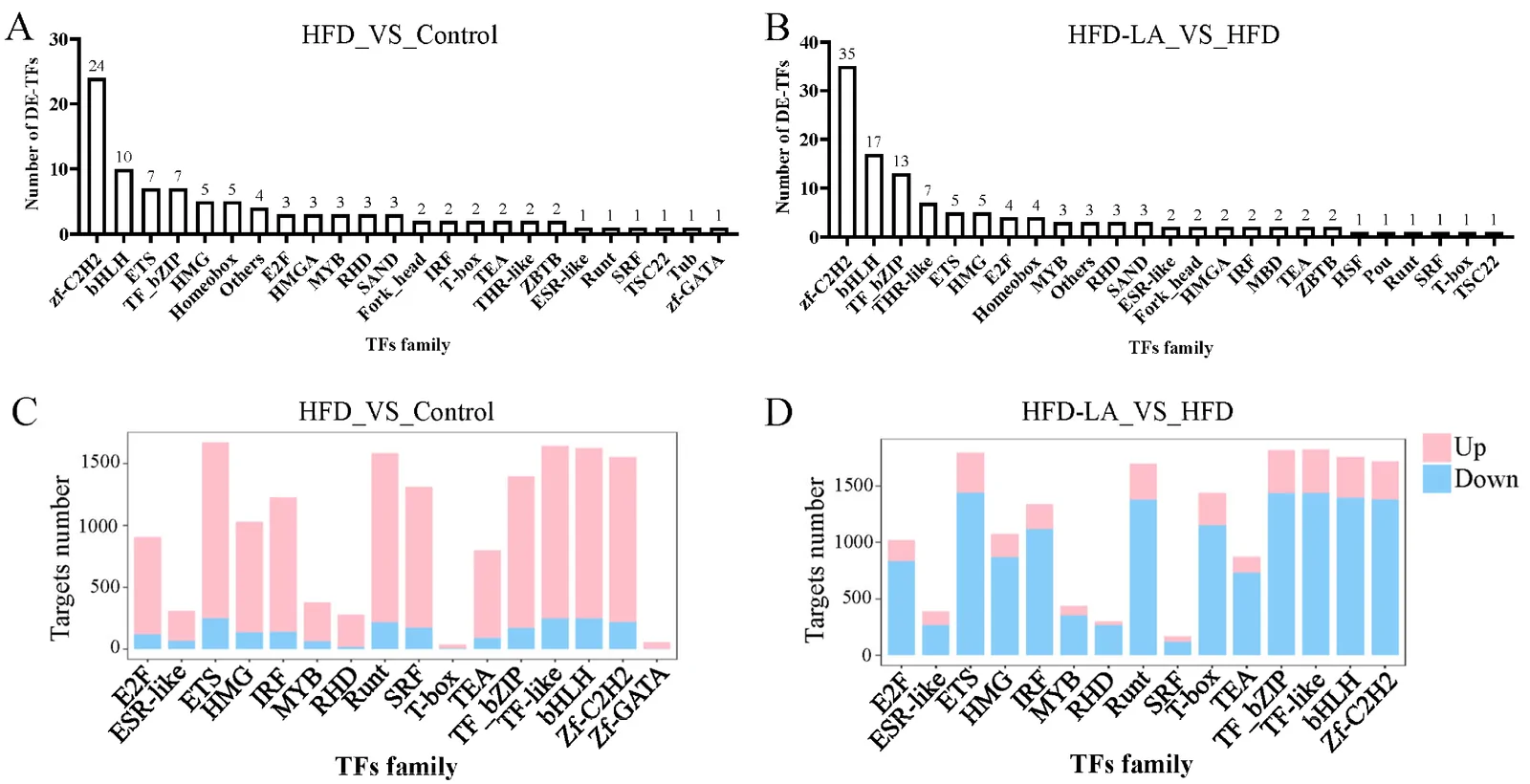
Statistics of DE-TF family numbers. (**A**) Number of DE-TF families in HFD_VS_Control. (**B**) Number of DE-TF families in HFD-LA_VS_HFD. (**C**) Counts of computationally predicted target genes of DE-TF families in HFD_VS_Control. (**D**) Counts of computationally predicted target genes of DE-TF families in HFD-LA_VS_HFD.

In addition, we profiled the DE-TFs to classify them systematically. All DE-TFs were classified into 26 families, among which the zf-C2H2 family was dominant, followed by the bHLH, TF-bZIP, and ETS families (Figure 3A,B). Analysis of the target genes of DE-TFs revealed that most were upregulated in HFD_VS_Control, whereas the opposite trend was observed in HFD-LA_VS_HFD (Figure 3C,D).

### 3.4. Function Enrichment Analysis of DE-TFs

**Figure 4:**
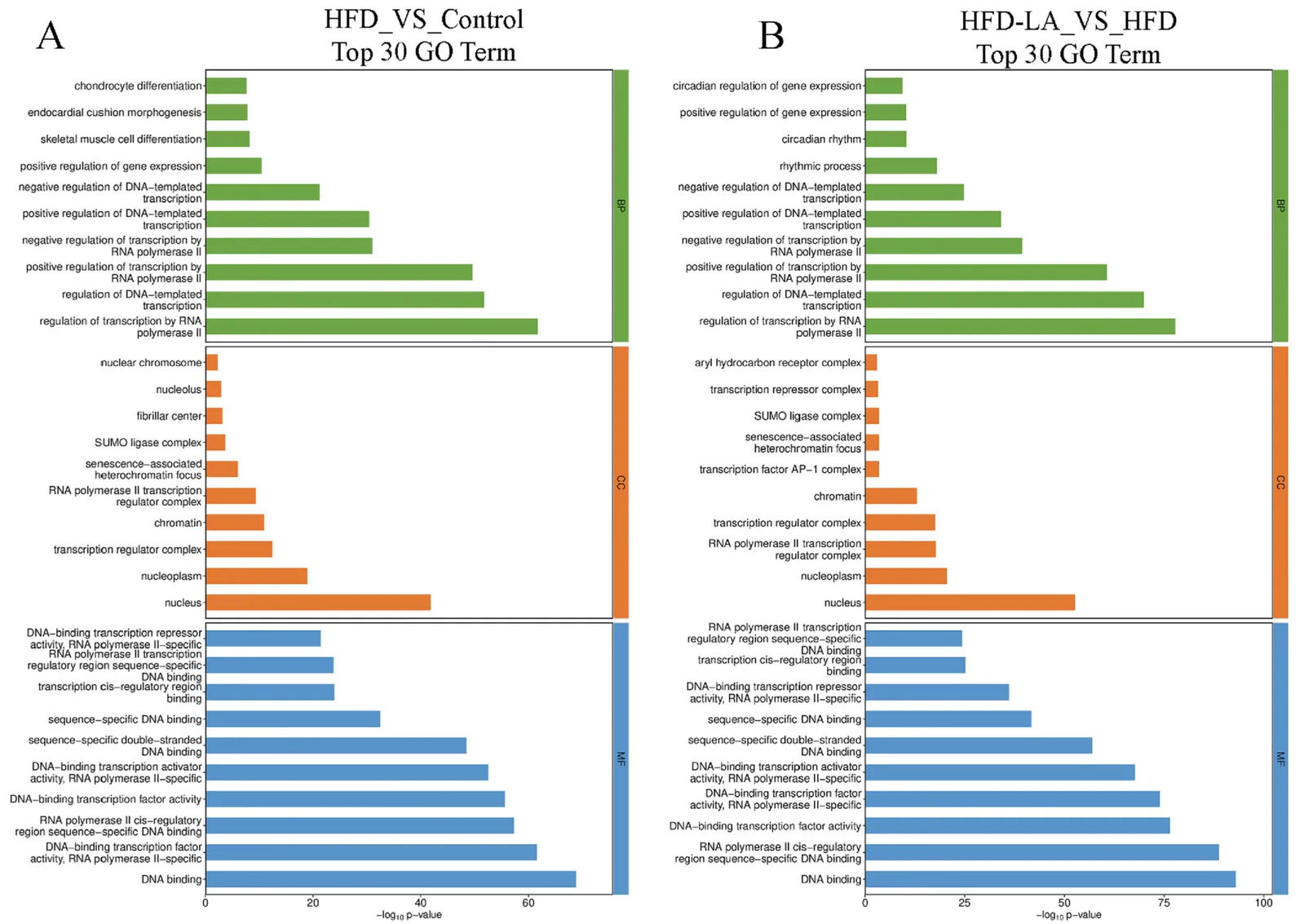
GO enrichment of DE-TFs. (**A**) Top 30 significantly enriched GO terms for DE-TFs in HFD_VS_Control. (**B**) Top 30 significantly enriched GO terms for DE-TFs in HFD-LA_VS_HFD. A *q*-value < 0.05 and |log_2_FC| ≥ 1 were considered to be significant.

GO enrichment analysis of DE-TFs revealed that 225 and 237 terms were significantly enriched in HFD_VS_Control and HFD-LA_VS_HFD, respectively (Supplementary Table S4). The top 30 most significantly enriched GO terms are shown in Figure 4. In both the HFD_VS_Control and HFD-LA_VS_HFD comparisons, regulation of transcription by RNA polymerase II (GO:0006357), nucleus (GO:0005634), and DNA binding (GO:0003677) were the most significantly enriched terms in biological process (BP), cellular component (CC) and molecular function (MF), respectively (Figure 4).

**Figure 5:**
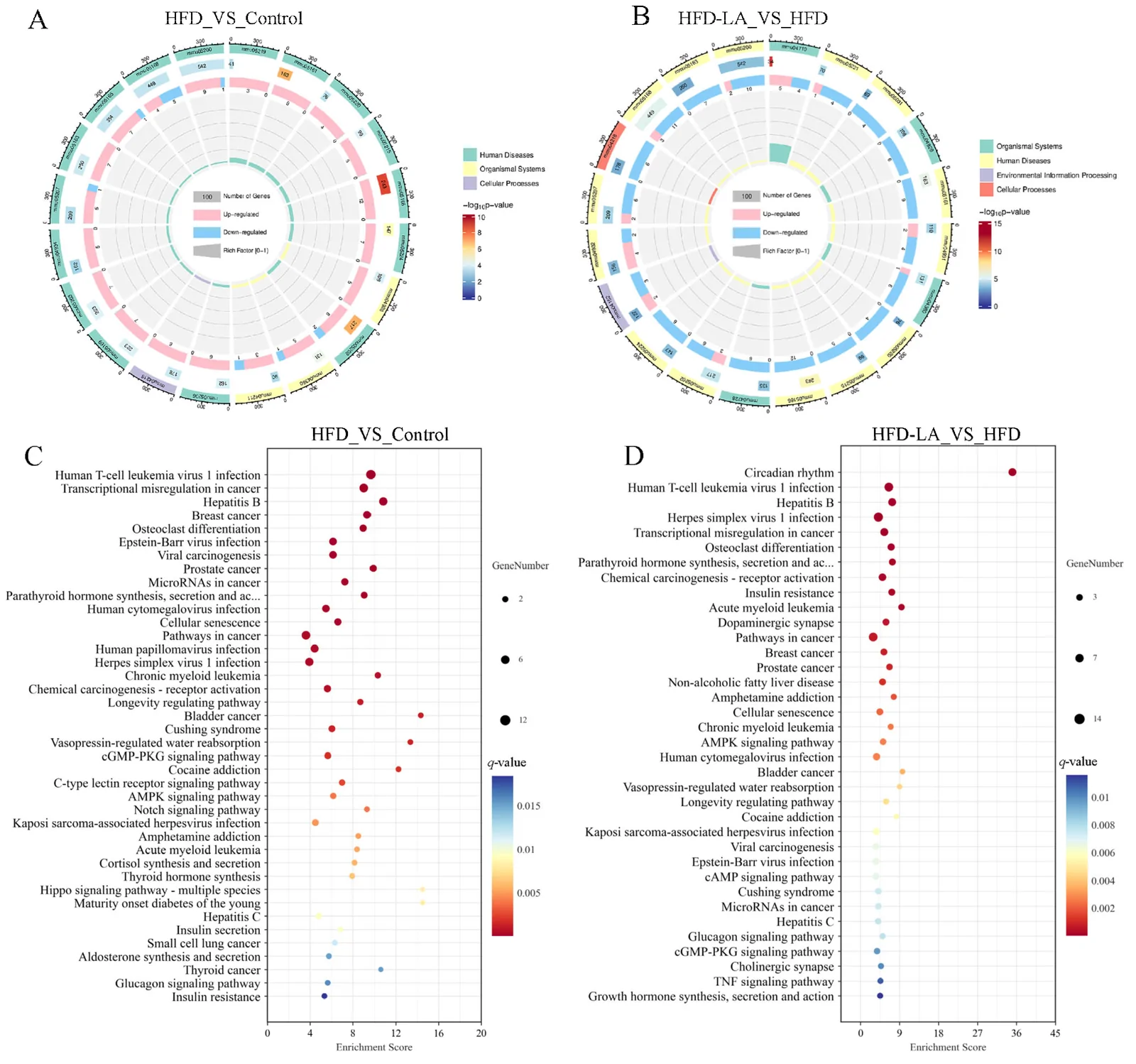
KEGG pathway enrichment of DE-TFs. (**A**) Circus plot of KEGG pathway enrichment for DE-TFs in HFD_VS_Control. (**B**) Circus plot of KEGG pathway enrichment for DE-TFs in HFD-LA_VS_HFD. (**C**) KEGG pathway enrichment of DE-TFs in HFD_VS_Control. (**D**) KEGG pathway enrichment of DE-TFs in HFD-LA_VS_HFD. A *q*-value < 0.05 and |log_2_FC| ≥ 1 were considered to be significant.

KEGG analysis showed that 40 and 36 pathways were significantly enriched in HFD_VS_Control and HFD-LA_VS_HFD, respectively (Supplementary Table S5). According to the primary classification, these pathways were mainly assigned to three categories: human diseases, organismal systems, and cellular processes (Figure 5A,B). Further subclassification demonstrated that the annotated pathways were primarily involved in multiple functional modules, including cancer and cancer-specific types (e.g., pathways in cancer, transcriptional mis-regulation in cancer), viral infectious diseases (e.g., human T-cell leukemia virus 1 infection, hepatitis B, human cytomegalovirus infection), signal transduction (e.g., AMPK signaling pathway, cGMP-PKG signaling pathway), as well as endocrine and metabolic disease (e.g., insulin resistance, MASLD, Cushing syndrome, maturity onset diabetes of the young) (Figure 5C,D). Notably, cancer- and viral-infection-related KEGG terms likely arise from the annotation bias of these pleiotropic transcription factors and do not indicate oncogenic or viral events in our HFD-induced MASLD model. We focus on metabolism- and inflammation-related pathways for biological interpretation.

### 3.5. PPI Network Construction and Analysis

**Figure 6:**
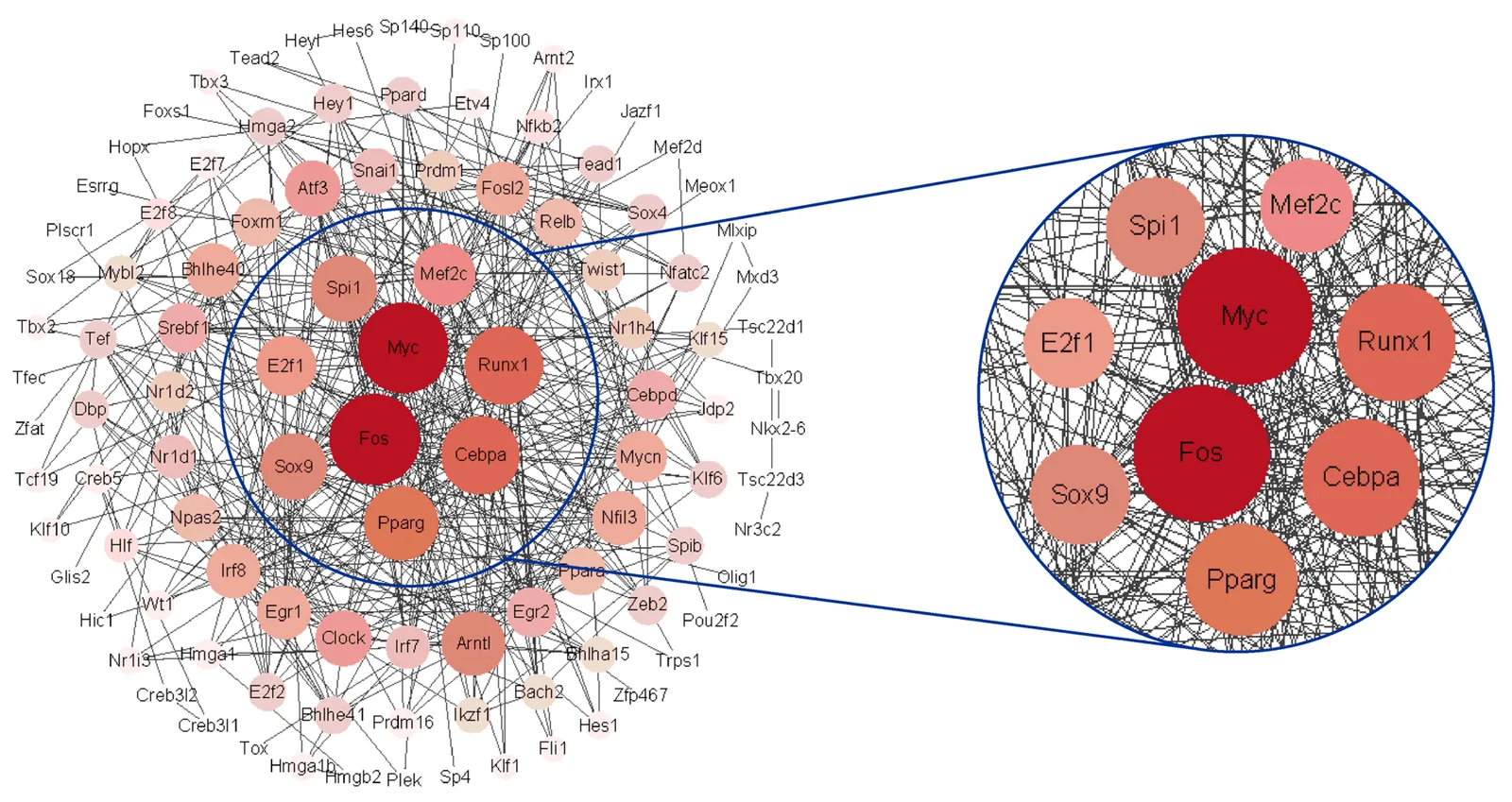
Screening of hub DE-TFs through protein–protein interaction network (PPI) analysis.

**Figure 7:**
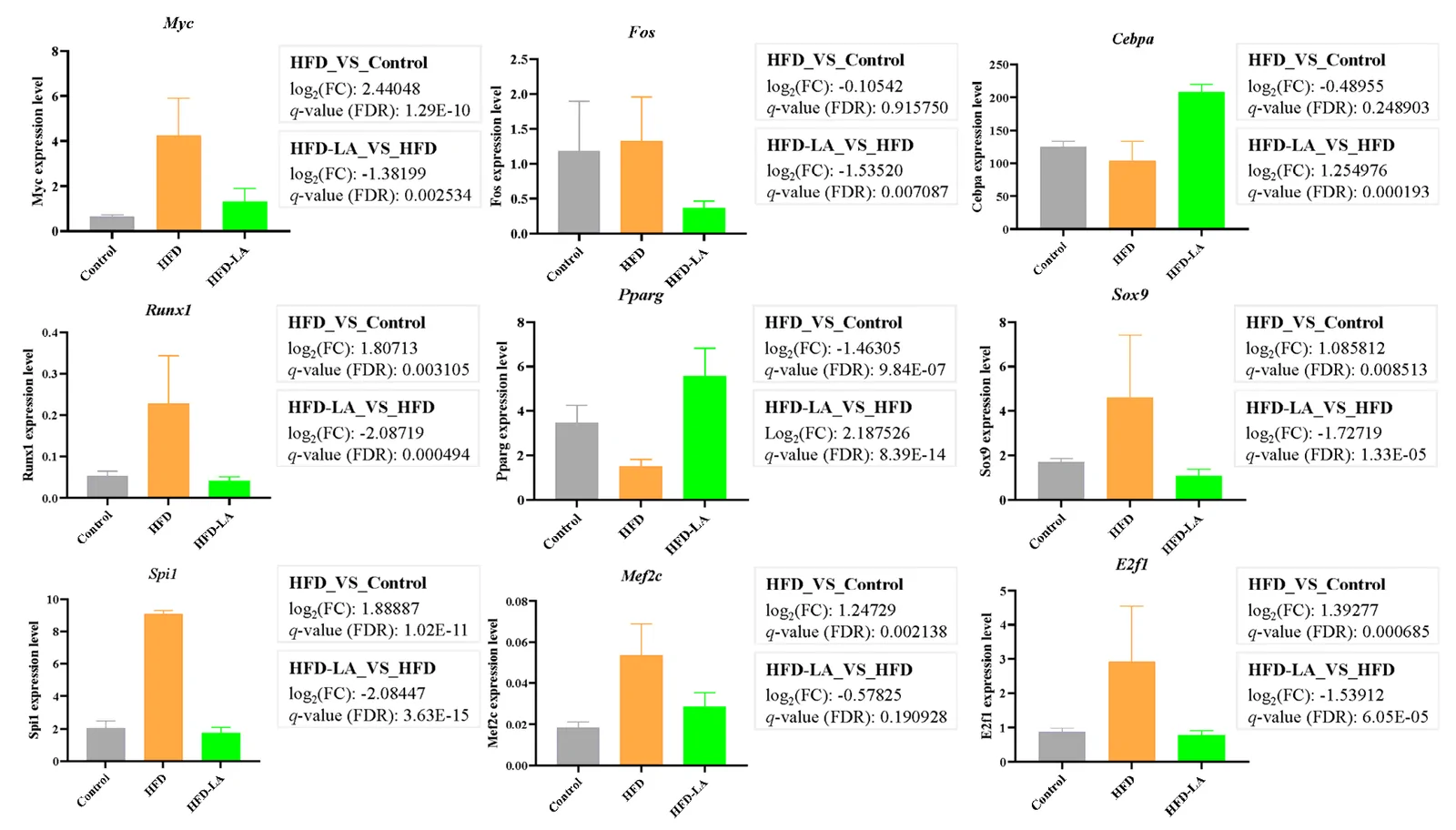
RNA-seq expression of nine hub transcription factors (*n* = 3). Differential expression was determined by DESeq2 using negative binomial test. log_2_(FC) and FDR *q*-values for HFD_VS_Control and HFD-LA_VS_HFD are shown.

To screen candidate hub TFs from DE-TFs, the PPI network was constructed using STRING and Cytoscape software based on protein–protein interaction topology. Nine candidate hub TFs were obtained according to the above-mentioned screening criteria, including *Myc*, *Fos*, *Cebpa*, *Runx1*, *Pparg*, *Mef2c*, *Spi1*, *Sox9*, and *E2f1* (Figure 6). Compared with the Control group, the expression levels of *Myc*, *Fos*, *Runx1*, *Mef2c*, *Spi1*, *Sox9* and *E2f1* were significantly upregulated, while the expression levels of *Cebpa* and *Pparg* were downregulated in the HFD group. Notably, LA treatment partially counteracted HFD-driven alterations in the transcript levels of these hub TFs (Figure 7).

**Figure 8:**
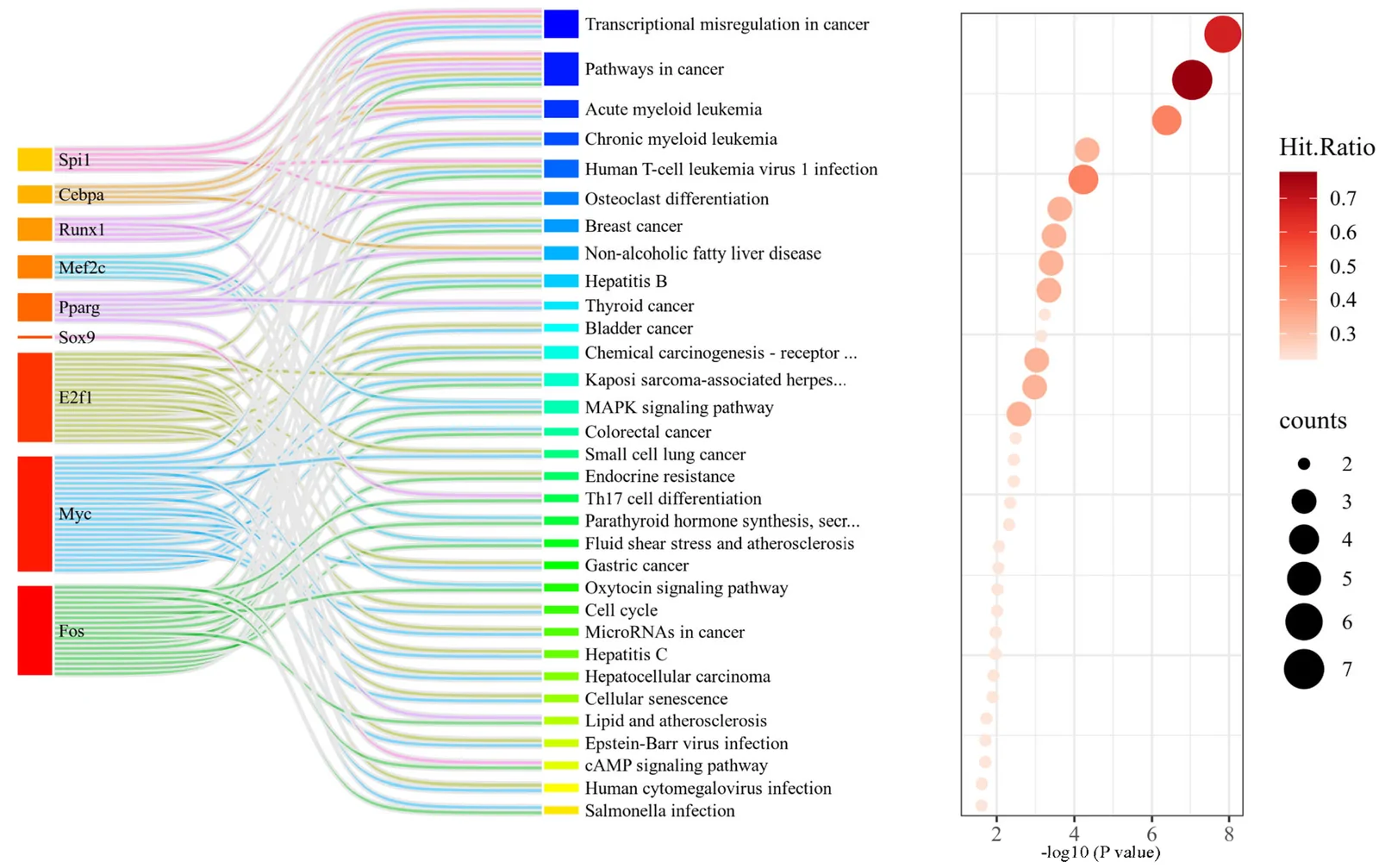
Sankey plot of enriched KEGG pathways for hub TFs.

Additionally, KEGG enrichment analysis was performed to investigate the hub TFs. We found that all hub TFs except *Sox9* were significantly enriched in the category of cancer and cancer-specific types, e.g., transcriptional mis-regulation in cancer, pathways in cancer, acute myeloid leukemia, chronic myeloid leukemia, and hepatocellular carcinoma. *E2f1*, *Fos*, and *Myc* were markedly enriched in viral infectious diseases, e.g., human T-cell leukemia virus 1 infection and hepatitis B. *Fos*, *Mef2c*, *Myc*, and *Sox9* were associated with signal transduction pathways, e.g., the MAPK signaling pathway, cAMP signaling pathway, and oxytocin signaling pathway. In addition, *Cebpa*, *Fos*, *Pparg*, and *Mef2c* were significantly enriched in endocrine and metabolic diseases, e.g., MASLD, lipid and atherosclerosis, and fluid shear stress and atherosclerosis (Figure 8).

### 3.6. Validation of the Hub TFs by RT-qPCR

**Figure 9:**
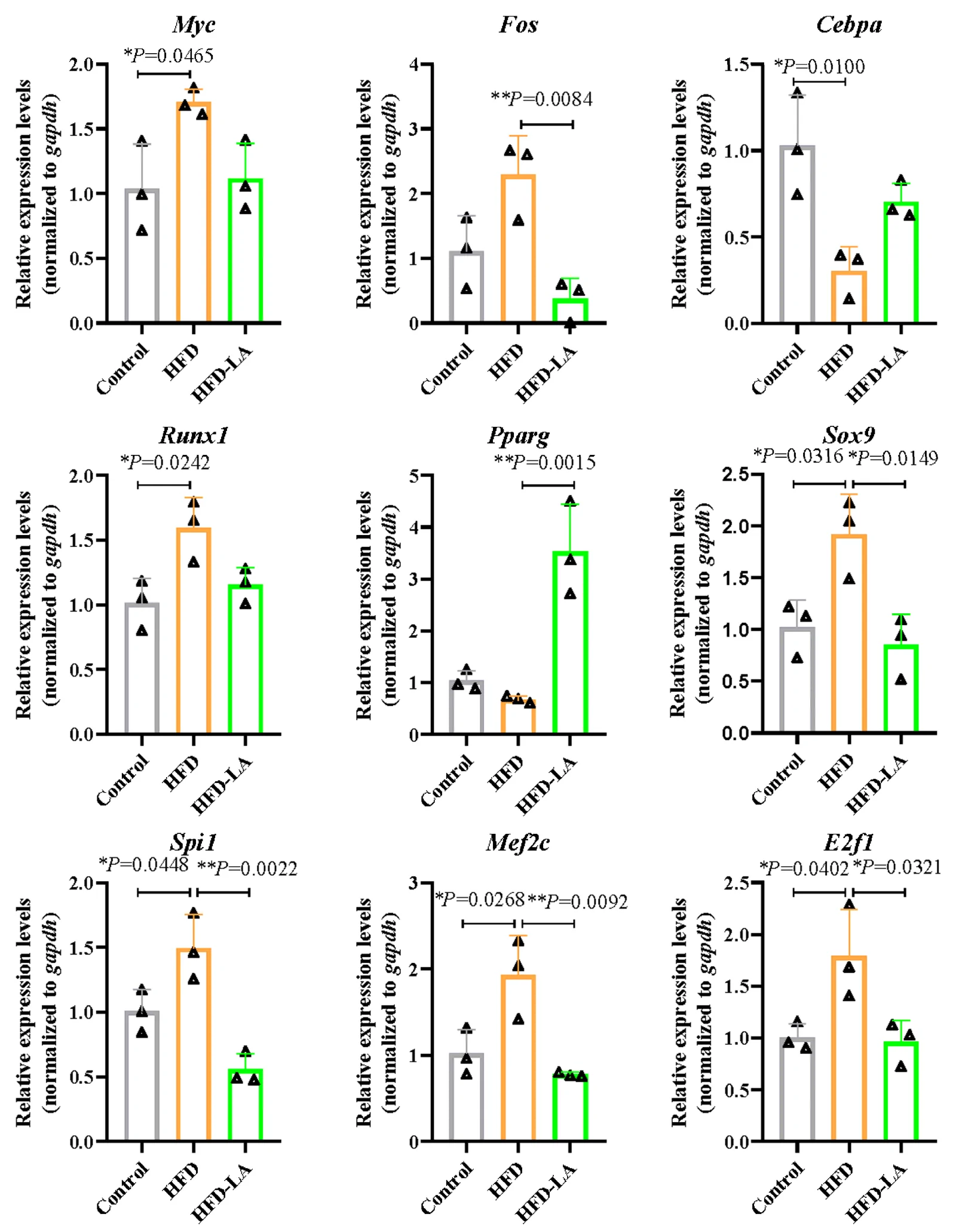
mRNA expression analysis of nine hub DE-TF genes by RT-qPCR. RT-qPCR was performed on liver RNA isolated from an independent cohort of MASLD mice separate from those used for RNA-seq (*n* = 3). Statistical analysis was performed by ordinary one-way ANOVA followed by Tukey’s multiple comparisons test. * *p* < 0.05, ** *p* < 0.01.

To cross-validate the mRNA expression patterns of the nine screened hub TFs identified by RNA-seq, RT-qPCR was performed on liver tissues obtained from an independent cohort of MASLD mice. The mRNA expression trends of these nine hub TFs showed directional concordance with the RNA-seq results. Although all genes exhibited consistent up- or down-regulation directions, not all of these concordant changes reached statistical significance in the RT-qPCR assay (Figure 9). Notably, this mRNA-level validation confirms transcriptional changes only and cannot directly prove the biological function or causal mechanistic roles of these hub transcription factors.

## 4. Discussion

TFs serve as crucial mediators that tightly regulate gene expression programs in cells. Their dysfunction disrupts transcriptional networks, contributing to the development of numerous diseases such as cancer, diabetes and obesity [13]. In this study, RNA-seq revealed 95 abnormally dysregulated TFs under HFD-induced MASLD conditions in mice. Of the 68 shared transcription factors, LA intervention reversed the dysregulated expression of 67, and LA also modulated an additional 54 unique DE-TFs. Collectively, altered transcript levels of these TFs correlate with LA-mediated amelioration of MASLD-related hepatic phenotypes in this animal model. DEG patterns reflected tissue-specific molecular alterations in mouse liver. Furthermore, this study revealed that the Zf-C2H2 family represented the most abundant TF family altered during MASLD progression and upon LA-mediated amelioration. Its members, including *Egr-1* and *Egr-2*, are positively associated with liver fibrosis [14,15]; *Ikzf1* and *Zfp57* are positively correlated with liver carcinogenesis [16,17]; and *Klf6* alleviates hepatic lipid accumulation [18]. In our transcriptomic analysis, all these factors are upregulated during MASLD progression but display the opposite expression pattern following LA treatment.

Functional enrichment analysis revealed that the GO terms were mainly associated with RNA polymerase II, nucleus, and DNA binding, indicating that transcriptional programs relying on these components participate in MASLD pathogenesis in mice. KEGG enrichment analysis revealed that the DE-TFs were mainly enriched in the AMPK signaling pathway and cGMP-PKG signaling pathway. Previous studies have revealed that natural plant-derived bioactive components (e.g., rutin, pterostilbene, quercetin, buddleoside, berberine) and clinical drugs (e.g., liraglutide, metformin) ameliorate MASLD-related disturbances via the AMPK signaling pathway [19,20,21,22,23,24]. The cGMP-PKG pathway is closely linked to HSC activation, a core step driving liver-fibrosis development [25,26]. Transcriptomic enrichment analysis showed that DE-TFs were enriched in the AMPK and cGMP-PKG signaling pathways. It should be noted that this enrichment result is only derived from transcriptome profiling and does not represent actual functional activation of these pathways. Further experiments, such as detecting the phosphorylation status of key signaling proteins, are needed to verify whether LA alleviates HFD-induced hepatic injury by regulating the AMPK and cGMP-PKG signaling cascades.

Notably, a total of nine hub TFs were identified in this study. *Myc*, *Fos*, *Runx1*, *Mef2c*, *Spi1*, *Sox9* and *E2f1* were upregulated, while *Cebpa* and *Pparg* were downregulated in the MASLD mouse model. Concurrent LA intervention mitigated these disease-associated expression patterns, implying that these hub TFs may contribute to LA-driven hepatoprotection against the development of HFD-induced MASLD-like alterations in mice. As a multifunctional proto-oncogene, MYC facilitates de novo ceramide synthesis by modulating its target gene *Cers4*. Studies have demonstrated that intestine-specific reduction in MYC in mice relieves obesity, insulin resistance, hepatic steatosis and steatohepatitis induced by a HFD [27]. Increased *Myc* expression in the liver promotes the development of alcoholic liver disease by suppressing p53. Conversely, *Myc* inactivation or drug inhibition exerts preventive and reversing effects on fatty liver disease. Therefore, *Myc* represents a potential biomarker and candidate target for fatty-liver-related research [28,29]. *Fos*, a key component of the AP-1 complex—a bZIP TF—has been implicated in insulin resistance, impaired lipid metabolism and chronic inflammation. Studies have shown that *Fos* drives the progression of T2DM-associated MASLD by transcriptional regulation of its downstream gene *Ptgs2* [30]. As a core modulator of liver function, *Cebpa* governs glucose and lipid homeostasis, myeloid cell differentiation and hepatocyte maturation. Meanwhile, it impedes liver fibrosis, mitigates liver damage and attenuates tumor burden in hepatocellular carcinoma [31,32]. *Runx1*, a new regulator of hepatic fibrosis, promotes hepatic stellate cell activation and liver fibrosis progression by regulating TGF-β signaling or *miR-24*/ALK4/Smad3 signaling [33,34]. *Pparg* modulates lipid metabolism and inflammation through regulating NF-κB signaling, and suppresses M1 macrophage polarization to alleviate MASLD [35,36]. *Sox9* is a promising research target for major hepatobiliary diseases and serves as a potential biomarker for fibrosis progression in MASLD [37]. Hepatocyte-specific *Sox9* knockout ameliorates acute liver injury by suppressing SHP signaling and improving mitochondrial function [38]. Pharmacological inhibition of *Spi1* restores glucose homeostasis and attenuates hepatic inflammation, steatosis and fibrosis [39]. In addition, *Mef2c* modulates hepatocellular carcinoma via the VEGF and Wnt/β-catenin signaling [40]. *E2f1*, an important regulator of metabolic diseases, promotes hepatic gluconeogenesis and sustained lipogenesis [41,42]. Importantly, it should be noted that high-connectivity nodes obtained from the STRING-PPI network represent topological candidates only. Topological connectivity cannot prove biological function or a causal mediation role. Our current evidence is limited to mRNA-level expression correlation from RNA-seq and RT-qPCR.

Given the chemical structure of LA, its biological effects are not limited to free-radical scavenging; it can also participate in the regulation of multiple intracellular signaling pathways [43]. In vitro experiments have confirmed that LA binds to proteins including PI3K, MEK1, and B-Raf to mediate biological functions, yet its direct molecular targets within the in-vivo hepatic microenvironment remain unclear. In light of published studies, the nine hub transcription factors identified in the present study represent valuable potential biomarkers and effector targets for MASLD research. Based on our transcriptomic data, we speculate that in the livers of MASLD mice, LA may act on upstream signaling molecules to reprogram downstream transcription-factor networks, thereby ameliorating hepatic lipid metabolic disorders.

Several limitations exist in this work. In the present study, LA was administered concurrently with HFD feeding; therefore, our findings reflect protective effects against MASLD development, rather than therapeutic reversal of pre-established MASLD lesions. Further studies using therapeutic regimens (LA treatment after MASLD onset) are warranted to explore its reversal potential. Additionally, parameters regarding body weight changes and food intake were not obtained in the present study. Our follow-up animal studies will track daily feed consumption and assess feed efficiency to parse the independent contributions of energy ingestion and energy expenditure. The expression changes in hub transcription factors were only validated at the mRNA level in mouse liver tissues, without cellular functional verification. Protein-level validation and functional assays are required to further dissect the biological roles of these hub transcription factors. Furthermore, hepatic transcriptome sequencing was conducted with three biological replicates per group, potentially raising the risk of false-positive DEGs. While RT-qPCR using three biological replicates confirmed the reliability of sequencing results, future studies with expanded sample sizes are needed to strengthen these experimental findings. Whether LA directly targets these hub transcription factor proteins remains unconfirmed, and indirect regulation via upstream signaling pathways is highly possible. In addition, only one dose of LA was used in animal experiments. Further cell-based functional assays and protein–ligand interaction analyses are required to clarify the detailed molecular mechanisms. This work is based solely on a mouse-model system; extrapolation to human MASLD requires considerable caution and further investigations.

## 5. Conclusions

In summary, this study used RNA-seq profiling to identify DE-TFs linked to MASLD pathogenesis and LA-mediated hepatoprotection in a mouse HFD model. Network analysis further uncovered nine hub TFs: seven upregulated factors (*Myc*, *Fos*, *Runx1*, *Mef2c*, *Spi1*, *Sox9*, and *E2f1*) previously reported to promote MASLD progression, and two downregulated factors (*Cebpa* and *Pparg*) that exert protective roles against MASLD. Concurrent LA treatment mitigated disease-relevant dysregulation of these hub-TF transcripts, indicating their potential regulatory roles in HFD-driven MASLD development and LA-mediated hepatic protection. Additional molecular studies are needed to dissect the individual biological functions of each hub TF in MASLD development, including whether LA can reverse already-established MASLD lesions. Together, our results expand knowledge of TF-driven transcriptional regulation in HFD-induced MASLD in mice and provide a transcriptomic resource for future mechanistic research on LA-related hepatoprotective bioactivity.

## Figures and Tables

**Table 1:** A summary of the sequencing data.

Sample	Raw Reads (M)	Clean Reads (M)	Valid Bases (%)	Q30 (%)	GC (%)	Mapped Reads (%)
Control 1	24.43	23.89	97.8	97.83	48.21	99.29
Control 2	23.81	23.35	98.1	97.76	47.86	99.08
Control 3	24.48	23.78	97.14	97.86	48.12	99.09
HFD 1	24.74	23.85	96.4	97.94	49.09	99.53
HFD 2	24.87	24.17	97.17	97.58	49.07	99.52
HFD 3	24.64	23.94	97.17	97.64	49.37	99.56
HFD-LA 1	24.89	23.97	96.29	97.68	48.16	99.52
HFD-LA 2	24.54	23.97	97.68	97.76	48.3	99.34
HFD-LA 3	24.46	23.76	97.12	98.1	48.78	99.59

## Data Availability

The original data presented in the study are openly available in the National Center for Biotechnology Information (NCBI) at BioProject ID PRJNA1478173.

## Supplementary Materials

The following supporting information can be downloaded at: https://www.mdpi.com/article/10.3390/nu18213564/s1, Table S1: Software parameters and reference genome information; Table S2: Primers used for RT-qPCR analysis; Table S3: Identification of DE-TFs in HFD_VS_Control, HFD-LA_VS_HFD, and 68 DE-TFs. A *q*-value < 0.05 and |log_2_FC| ≥ 1 were considered to be significant; Table S4: GO enrichment of DE-TFs in HFD_VS_Control and HFD-LA_VS_HFD. A *q*-value < 0.05 was considered to be significant; Table S5: KEGG pathway enrichment of DE-TFs in HFD_VS_Control and HFD-LA_VS_HFD. A *q*-value < 0.05 was considered to be significant; Figure S1: Gene expression analysis. (**A**) FPKM distribution. (**B**) Sample correlation heatmap; Figure S2: Hierarchical clustering heatmaps of DE-TFs in mouse liver tissues for HFD_VS_Control (**A**) and HFD-LA_VS_HFD (**B**).

## Abbreviations

The following abbreviations are used in this manuscript:

BP	Biological Process
CC	Cellular Component
FPKM	Fragments Per Kilobase of Transcript Per Million Fragments Mapped
GO	Gene Ontology
HFD	High-Fat Diet
KEGG	Kyoto Encyclopedia of Genes and Genomes
LA	Licochalcone A
MASLD	Metabolic Dysfunction-Associated Steatotic Liver Disease
MF	Molecular Function
NAFLD	Nonalcoholic Fatty Liver Disease
PPI	Protein–Protein Interaction Network
RIN	RNA Integrity Number
RNA-seq	RNA-Sequencing
SPF	Specific-Pathogen-Free
TFs	Transcription Factors
